# The Predictive Performance of Risk Scores for the Outcome of COVID-19 in a 2-Year Swiss Cohort

**DOI:** 10.3390/biomedicines12081702

**Published:** 2024-07-31

**Authors:** Maria Boesing, Giorgia Lüthi-Corridori, David Büttiker, Mireille Hunziker, Fabienne Jaun, Ugne Vaskyte, Michael Brändle, Jörg D. Leuppi

**Affiliations:** 1 University Institute of Internal Medicine, Cantonal Hospital Baselland, 4410 Liestal, Switzerland; 2 Faculty of Medicine, University of Basel, 4056 Basel, Switzerland; 3 Department of Internal Medicine, Cantonal Hospital Sankt Gallen, 9000 Sankt Gallen, Switzerland

**Keywords:** COVID-19, SARS-CoV-2, outcome, severe course, in-hospital death, ventilation, risk score, COVID-COMBI

## Abstract

Various scoring systems are available for COVID-19 risk stratification. This study aimed to validate their performance in predicting severe COVID-19 course in a large, heterogeneous Swiss cohort. Scores like the National Early Warning Score (NEWS), CURB-65, 4C mortality score (4C), Spanish Society of Infectious Diseases and Clinical Microbiology score (COVID-SEIMC), and COVID Intubation Risk Score (COVID-IRS) were assessed in patients hospitalized for COVID-19 in 2020 and 2021. Predictive accuracy for severe course (defined as all-cause in-hospital death or invasive mechanical ventilation (IMV)) was evaluated using receiver operating characteristic curves and the area under the curve (AUC). The new ‘COVID-COMBI’ score, combining parameters from the top two scores, was also validated. This study included 1,051 patients (mean age 65 years, 60% male), with 162 (15%) experiencing severe course. Among the established scores, 4C had the best accuracy for predicting severe course (AUC 0.76), followed by COVID-IRS (AUC 0.72). COVID-COMBI showed significantly higher accuracy than all established scores (AUC 0.79, *p* = 0.001). For predicting in-hospital death, 4C performed best (AUC 0.83), and, for IMV, COVID-IRS performed best (AUC 0.78). The 4C and COVID-IRS scores were robust predictors of severe COVID-19 course, while the new COVID-COMBI showed significantly improved accuracy but requires further validation.

## 1. Introduction

COVID-19, caused by the virus SARS-CoV-2 and primarily affecting the respiratory system, caused a global pandemic, with its origin in Wuhan, China [1]. The World Health Organization declared a public health emergency of international concern in January 2020, which lasted until May 2023 [2,3,4]. While, nowadays, most infected individuals experience asymptomatic or mild to moderate COVID-19, courses with the need for hospitalization are still prevalent and severity ranges from the need for simple oxygen supplementation to Acute Respiratory Distress Syndrome (ARDS), respiratory failure, and death [5,6]. The severe course of COVID-19 requires immediate medical attention and intensive care management [5]. In addition to pharmacological therapy, effective management includes supportive care and mechanical ventilation [7]. Despite the advances in treatment options, changes in the virulence of SARS-CoV-2 variants, and the comprehensive availability of vaccines, recently reported in-hospital mortality for COVID-19 still ranges from 6% to 14%, depending on the population and virus variant studied [8,9].

Considerable research on risk factors for a severe course has been conducted since the outbreak of the COVID-19 pandemic in early 2020. Certain comorbidities (cardiovascular disease, diabetes, obesity), biomarkers (elevated interleukin-6, C-reactive protein (CRP), D-dimers), and radiological findings (pleural effusions, large extent of parenchymal lesions) have been reported to be associated with a severe course or death due to COVID-19 [10,11,12,13,14,15]. In clinical practice, several pre-existing scoring systems have been validated and used for COVID-19 risk stratification, including the qSOFA, NEWS, and CURB-65 scores [16,17,18,19,20,21]. Additionally, the urgent need for improved risk stratification resulted in the adaptation and new design of tools to specifically predict the severity and outcomes of COVID-19, such as the 4C mortality score, COVID-SEIMC score, and COVID-IRS score [22,23,24]. The mentioned scores differ widely in the respective utilization of parameters, ranging from demographic data and patient history via vital signs and symptoms to laboratory values [16,18,21,22,23,24,25,26] (see Table 1). The heterogeneity of parameters used in the established scores is a reflection of the diversity of risk factors for a severe course of COVID-19 and its multifactorial causes.

The listed prediction models were either not specifically designed to predict the outcome of COVID-19 or were built based on data from early 2020, when COVID-19 treatment options were limited and vaccinations were not yet available [22,23,24]. Furthermore, data used for the development of these risk scores were mostly from small cohorts of 100 to 200 patients. External validation and comparison of the established scores in a large, heterogeneous Swiss cohort are still missing to date.

When aiming to predict a severe course of COVID-19, it is important to take into account that the implementation of invasive mechanical ventilation is always an individual decision that is not dependent only on objective indication. A high proportion of patients or their relatives do explicitly decide against such an invasive step, regardless of the acuteness of their respiratory situation [27]. Thus, when mechanical ventilation is clinically indicated, individual patients’ beliefs and circumstances play an important role in the respective outcome. Consequently, ‘in-hospital death’ and ‘invasive mechanical ventilation’ should not be separated in terms of severe outcomes because both could indicate the same level of severity.

Even though the SARS-CoV-2 pandemic has strongly subsided, the identification of risk factors and the prediction of a severe course remain important. The early identification of individuals at high risk can prevent the progression of COVID-19 to ARDS by allowing physicians to initiate prompt interventions and appropriate treatment strategies. Additionally, the prediction of a severe course can provide valuable prognostic information for healthcare providers, affected patients, and their relatives. Finally, the identification of risk factors for a severe course of COVID-19 helps define suitable eligibility criteria for clinical trials further investigating preventive and therapeutic options for COVID-19.

### Objectives

This study aimed to externally validate different available scoring systems in a large, heterogeneous Swiss cohort and compare their predictive accuracy for a severe course of COVID-19. A secondary aim was the validation of a prediction model that combines parameters used in the two best-performing scores.

## 2. Materials and Methods

### 2.1. Study Design and Setting

This project was a retrospective, observational, single-center study. Adult patients who were hospitalized for COVID-19 for at least one night at the Cantonal Hospital Baselland, Switzerland (KSBL), between March 2020 and December 2021 and fulfilling the eligibility criteria (see Section 2.2) were included in this study. The KSBL is a public teaching hospital providing medical care for a population of approximately 250,000.

### 2.2. Study Population

Adult patients (18 years or older) who were hospitalized for COVID-19 as their main diagnosis for at least one night at the KSBL were eligible for inclusion in this study. Patients who were transferred to the KSBL from another acute care hospital or declined the hospital’s general research consent were excluded. In cases where a patient was hospitalized multiple times for COVID-19 within the given period, only the first hospitalization was included in the data collection to avoid bias.

### 2.3. Outcomes and Scores

The primary outcome was a severe course of COVID-19, defined as the composite of either in-hospital death or invasive mechanical ventilation. The outcomes of secondary interest were the individual endpoints in-hospital death and invasive mechanical ventilation. In-hospital death was defined as all-cause death during the hospitalization of interest at KSBL or during an immediate subsequent hospitalization in the case of transfer to a different acute care institution. Invasive mechanical ventilation was defined as endotracheal intubation, tracheostomy, or extracorporeal membrane oxygenation during the hospitalization of interest at KSBL or during an immediate subsequent hospitalization in the case of transfer to a different acute care institution.

The National Early Warning Score (NEWS), CURB-65 score (CURB-65), 4C mortality score (4C), Spanish Society of Infectious Diseases and Clinical Microbiology score (SEIMC), and COVID Intubation Risk Score (COVID-IRS) at the time of admission were retrospectively calculated for 1051 patients consecutively hospitalized for COVID-19 at KSBL in 2020 and 2021. Table 1 provides an overview of the scores that were compared in this study, together with their original purpose and the parameters used. Their respective formulas for calculation can be found in Appendix A, Table A1, Table A2, Table A3, Table A4, Table A5 and Table A6.

The above-mentioned scores were designed to predict either in-hospital death or mechanical ventilation. Since the severe course was a composite of these two outcomes, we defined a new score ‘COVID-COMBI’ as a combination of the best-performing scores for each outcome: 4C for in-hospital death and COVID-IRS for mechanical ventilation. The COVID-COMBI represents a plain summation of the two scores, except that the only common parameter, ‘respiratory rate’, was only weighted once, according to the rule in the COVID-IRS. The COVID-COMBI ranges between 0 and 32, with higher scores indicating a higher risk of a severe course. Table 2 presents the formula for the calculation of COVID-COMBI.

### 2.4. Data Collection and Management

After the verification of eligibility, patient outcomes and parameters for score calculations were manually extracted from the electronic health records. These included discharge reports, nursing documentation, emergency reports, intensive care unit (ICU) reports, laboratory records, and radiology findings. Data were entered into a REDCap^®^ (Research Electronic Data Capture) database.

Scores were calculated with parameters available at the time of admission or for laboratory values up to 24 h after admission. Specifically, relevant vital signs and symptoms and records of mental status were taken from the emergency department (ED) documentation (first documented in-house measurement). FiO_2_ was estimated from the oxygen supplementation flow rate by means of the Vincent formula [28,29]. For relevant laboratory values and radiological findings, the first in-house result up to 24 h after admission was used for the score calculations, when indicated. Information about relevant comorbidities and patient history was taken from documented anamnesis and previously documented diagnosis lists.

The data presented in this study are not publicly available due to restrictions in data privacy but are available upon reasonable request from the corresponding author.

### 2.5. Statistical Analysis

Patient data were analyzed descriptively and presented as absolute and relative frequencies or median and interquartile ranges (IQRs). For the score calculation, variables with missing values were imputed using the k-nearest neighbor algorithm [30]. As a sensitivity analysis, all validations were additionally performed on the original, non-imputed dataset.

Predictive performance for each outcome was compared by means of receiver operating characteristic (ROC) curves and the respective area under the curve (AUC) with 95% confidence intervals (95% CI). The resulting ROC curves were compared pairwise with a Z-test following DeLong’s method [31]. All reported *p*-values were two-sided at a significance level of 0.05. Data imputation and analysis were performed with R version 4.1.0 using the packages ‘bnstruct’, ‘rms’, and ‘pROC’ [32].

### 2.6. Ethical Considerations

This study was approved by the ethics committee of Northwestern and Central Switzerland (ENKZ, BASEC Project-ID 2022-01636, approved on 22 September 2022). Patients who declined consent to the further use of their clinical routine data for research purposes (general research consent) were excluded from this study.

## 3. Results

### 3.1. Patient Characteristics and Outcomes

Between March 2020 and December 2021, 1274 patients were hospitalized with a main diagnosis of COVID-19 for at least one night at the KSBL. The first hospitalization for COVID-19 at the KSBL occurred on 3 March 2020. After excluding 150 patients who denied consent for the use of their data for research purposes and 73 patients who were admitted from another hospital, 1051 patients were included in this study.

Table 3 summarizes the characteristics and outcomes of the included patients. The median age was 65 years, ranging from 19 to 99 years (IQR: (54, 79)), and 59.7% of the patients were male (*n* = 627). The most prevalent comorbidity was arterial hypertension (45.8%, *n* = 481), followed by obesity (31.0%, *n* = 286), diabetes (23.0%, *n* = 242), and chronic kidney disease (19.5%, *n* = 205). The majority of the included patients were hospitalized at a stage when vaccination was not yet available (68.8%, *n* = 615). This included patients who were hospitalized when vaccines were already approved in Switzerland but not yet recommended for their respective age and risk group. After the comprehensive availability of vaccines, almost two-thirds of the patients were still not vaccinated upon admission (63.8%, 178 out of 279). The most common COVID-19 symptom upon admission was coughing (68.5%, *n* = 715), while 46.2% were suffering from dyspnea (*n* = 483). New onset of confusion was rare (3.5%, *n* = 37). While median heart rate and body temperature were within the physiological range at presentation, 38.4% of patients were febrile with a body temperature ≥ 38 °C (*n* = 397). Median systolic blood pressure was slightly increased at 134 mmHg. The majority of patients (60.3%, *n* = 634) presented with a peripheral oxygen saturation (SpO_2_) ≥ 92% at room air. The remaining 39.7% (*n* = 417) either presented with a SpO_2_ < 92% at room air or were already supplemented with oxygen by the paramedics.

White blood cell count was predominantly within the normal range upon admission (median 6.1, IQR (4.5, 8.0)), but the majority of patients presented with an elevated NLR (median 5.1, IQR (3.1, 8.5)), indicating high levels of inflammation. Accordingly, elevated CRP, urea, and LDH values were common. The majority of patients presented with slightly reduced kidney function, with an eGFR < 90 mL/min/1.73m^2^ (69.9%, *n* = 719 out of 1029).

Out of the 1051 included patients, 162 patients experienced a severe course (15.4%). In total, 112 patients died (10.7%) and 74 patients were mechanically ventilated (7.0%) during their hospitalization. Out of the mechanically ventilated patients, 67.6% were discharged alive (*n* = 50). Table 4 summarizes the calculated scores of the overall population at admission and by outcome, respectively. Patients who suffered from a severe course and those who died in-hospital scored higher for all established scores, except qSOFA, compared to the overall population. Patients who required invasive mechanical ventilation scored higher than the overall population for NEWS, 4C, and COVID-IRS scores but not qSOFA, CURB-65, and COVID-SEIMC scores. qSOFA scores did not differ amongst the overall population and the sub-groups of patients with adverse outcomes. In the new COVID-COMBI score, patients with any of the three adverse outcomes presented with higher values than the overall population.

### 3.2. Prediction of Severe Course, In-Hospital Death, and Invasive Mechanical Ventilation

**Figure 1:**
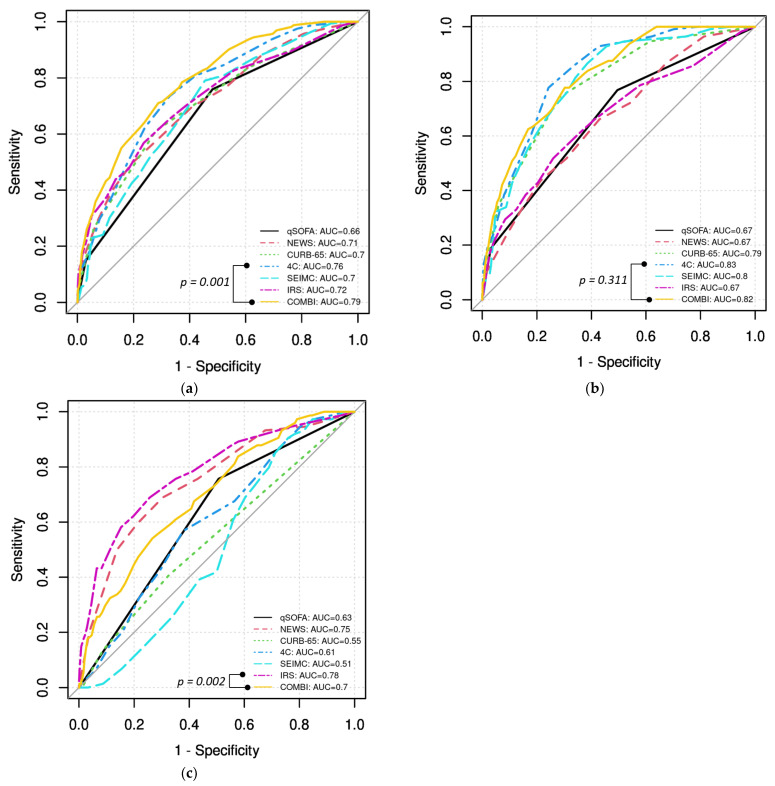
Receiver operator characteristic curves for the prediction of (**a**) severe course, (**b**) in-hospital death, and (**c**) invasive mechanical ventilation. Abbreviations: AUC: area under the curve. qSOFA: quick Sequential Organ Failure Assessment. NEWS: National Early Warning Score. 4C: 4C mortality score. SEIMC: Sociedad Española de Enfermedades Infecciosas y Microbiología Clínica (Spanish Society of Infectious Diseases and Clinical Microbiology). IRS: intubation risk score.

Predictive accuracy was assessed on the imputed dataset. Figure 1 presents the ROC curves of all analyzed scores for the prediction of the respective outcomes. From the established scores, 4C had the best accuracy in predicting severe course (AUC 0.76, 95% CI (0.72, 0.79)), followed by the COVID-IRS (AUC 0.72, 95% CI: (0.67, 0.76)). The new COVID-COMBI score showed significantly better accuracy to predict severe course, with an AUC 0.79 (95% CI: (0.75, 0.82), *p* = 0.001, see Figure 1a).

In-hospital death was also best predicted by the 4C score (AUC 0.83, 95% CI: (0.80, 0.86)), followed by the SEIMC score (AUC 0.80, 95% CI: (0.76, 0.84)). The new COVID-COMBI score reached a similar predictive accuracy to the 4C (AUC 0.82, 95% CI: (0.78, 0.86), *p* = 0.311) (see Figure 1b).

Invasive mechanical ventilation was best predicted by the COVID-IRS (AUC 0.78, 95% CI: (0.72, 0.84)), followed by NEWS (AUC 0.75, 95% CI: (0.69, 0.81)). The new COVID-COMBI score only reached an AUC of 0.70 for the prediction of invasive mechanical ventilation (95% CI: (0.64, 0.76), *p* = 0.002 (COVID-IRS vs. COVID-COMBI)) (see Figure 1c).

The predictive accuracy of the qSOFA score was poor for all analyzed outcomes (all AUC < 0.7).

## 4. Discussion

This study evaluated the accuracy of six scores—qSOFA, NEWS, CURB-65, 4C, COVID-SEIMC, and COVID-IRS—in predicting severe course, in-hospital death, and invasive mechanical ventilation for hospitalized COVID-19 patients. Additionally, a newly developed composite score, COVID-COMBI, which combined the parameters of the two best-performing scores (4C and COVID-IRS), was validated. Its predictive accuracy was compared with that of the established scores. Our study has three main findings:

1. The 4C and COVID-IRS both showed good accuracy for the prediction of severe course.
2. The new COVID-COMBI score showed significantly better performance than all other established scores in predicting severe course.
3. The new COVID-COMBI score showed good accuracy for the prediction of in-hospital death and invasive mechanical ventilation.

### 4.1. Predictive Accuracy of Established Scores

The population studied in this project was a large, heterogeneous cohort, with patients hospitalized for COVID-19 in Switzerland within a two-year period. The 4C mortality score, COVID-SEIMC, and COVID-IRS are specifically designed for COVID-19 risk stratification. Hence, it is not surprising that they performed well in the prediction of the outcomes they were designed for. In the prediction of in-hospital death, the 4C reached an excellent AUC of 0.83 in our cohort, followed by the COVID-SEIMC, with an AUC of 0.80. Previous studies reported similar AUCs between 0.79 and 0.85 for the 4C [24,33,34] and between 0.75 and 0.85 for the COVID-SEIMC [23,33,35]. The good predictive accuracies of 4C and COVID-SEIMC confirm older age, male sex, and comorbidities as risk factors for COVID-19-related death. However, indicators of the acuteness of the situation such as SpO_2_ and laboratory parameters urea, CRP, LDH, and NLR also seem to play an important role.

The COVID-IRS, on the other hand, reached a reasonable AUC of 0.78 for the prediction of invasive mechanical ventilation in our cohort, which is a lower predictive accuracy than that reported in previous studies. In an internal validation by Garcia-Gordillo et al., the COVID-IRS reached an AUC of 0.88 [22]. An external validation on a cohort of 285 patients hospitalized in Taiwan in May and June 2021 reported an AUC of 0.82 [35]. The higher AUC in the Taiwanese cohort could be explained by the fact that patients with a ‘do-not-intubate’ order were excluded from the study [35]. However, in our study, COVID-IRS still reached the highest predictive accuracy for invasive mechanical ventilation amongst the analyzed scores, followed by NEWS. This result highlights the strong predictive value of vital signs indicating acute respiratory status (high respiratory rate, low peripheral oxygen saturation, or high SaO_2_/FiO_2_ ratio) and biomarkers of inflammation (LDH, NLR) for intubation, which has been previously reported [36,37,38,39,40].

Overall, in view of the different setting and the heterogeneity of our cohort, these results indicate the 4C’s and COVID-IRS’s robustness against changing virulence, treatment options, and vaccination status. Notably, the NEWS score showed moderate predictive accuracy for invasive mechanical ventilation (AUC 0.75), aligning with its intended purpose of detecting early clinical deterioration and previous results [6,17,41].

The six different scores were designed for different populations and different endpoints; hence, their direct comparison is tentative. However, previous studies reported good predictive accuracy of scores for COVID-19 outcomes they were not designed for [16,18,20]. The results of this study confirm that scores such as the NEWS, although developed for a different outcome, feasibly compete with COVID-IRS and 4C in the prediction of mechanical ventilation and in-hospital mortality, respectively. Especially when seeking to predict the composite outcome ‘severe course’, it is pragmatic to search for predictive parameters within those that predict the respective single outcome.

When examining the composite endpoint ‘severe course’, the prediction accuracy of both 4C and COVID-IRS remained acceptable, with AUCs of 0.76 and 0.72, respectively. This result was rather unexpected since the two scores are based on a completely different set of parameters: While the 4C takes demographics (age, sex) and comorbidities into account, the COVID-IRS focuses solely on vital signs and laboratory values (Table 1). The only parameter used by both scores is the patient’s respiratory rate upon admission. This observation suggested that a combination of these factors could further improve prediction accuracy.

Interestingly, the qSOFA did not effectively predict any of the adverse outcomes (AUC < 0.7 for all), which suggests limited utility in the COVID-19 patient population. This finding is consistent with previous studies, indicating that it may not be suitable for predicting severe outcomes in COVID-19 patients [33,41,42].

### 4.2. Predictive Accuracy of COVID-COMBI

The validation of COVID-COMBI (Table 2), combining elements of both the 4C and COVID-IRS, confirmed the above-stated hypothesis: The COVID-COMBI score reached significantly better prediction accuracy than all other assessed scores, with an AUC of 0.79. This result suggests that a severe course of COVID-19 is often influenced by multiple factors and, therefore, can only be reliably predicted by a combination of pre-existing risk factors, indicators of acute respiratory status, and inflammatory markers. Furthermore, the prediction accuracy of COVID-COMBI for in-hospital death with an AUC of 0.82 was just marginally worse than that of the 4C. On the other hand, in the prediction of mechanical ventilation, COVID-COMBI performed significantly worse than the COVID-IRS, even though it incorporates all parameters also used in the COVID-IRS. This finding may reflect the complexity of predicting mechanical ventilation, which depends not only on clinical severity but also on non-intubation decisions. As reported multiple times before, older patients and those with a high number of comorbidities are at high risk for a severe course of COVID-19 [11,12,13,43]. At the same time, these patients are particularly likely not to be mechanically ventilated in case of respiratory deterioration, either due to a poor individual prognosis or their personal beliefs and life circumstances [44]. This relationship may result in poor predictive power of age and number of comorbidities for mechanical ventilation or even a negative association.

With an AUC of 0.79, the accuracy of COVID-COMBI for the prediction of severe course can be classified as moderate to good. It is important to keep in mind that the COVID-COMBI is a plain combination of previously validated scoring systems. Predictive accuracy could possibly be further improved by including other reportedly strong predictors that are routinely available in the scoring system, such as COVID-19 vaccination status, immunosuppression, and obesity [45,46,47,48].

### 4.3. Limitations

Our study has several limitations. First, the single-center, retrospective design with a population of only 1051 patients may limit the generalizability of its results. The unavailability of certain parameters in the clinical routine data prevented us from calculating other relevant scores. Missing values in some parameters, despite being imputed, might have introduced some bias; however, sensitivity analyses of the non-imputed dataset yielded consistent findings. Finally, we did not stratify our cohort by virus variant, vaccination status, or pharmacological treatment due to the small size of the subsets. On an even larger cohort, this could potentially enhance the accuracy of the results.

## 5. Conclusions

Our study demonstrates that both the 4C mortality score and the COVID-IRS score have robust predictive accuracy for the prediction of severe course in patients hospitalized for COVID-19. Notably, the newly developed COVID-COMBI score outperformed both scores in the prediction of severe disease progression. Additionally, the COVID-COMBI score performed well in the prediction of in-hospital death and invasive mechanical ventilation. The findings from this study highlight the potential utility of the COVID-COMBI score in clinical practice. By integrating comprehensive risk factors and clinical routine parameters, the COVID-COMBI can help healthcare providers identify high-risk patients early, enabling timely interventions and potentially improving patient outcomes. The score’s robust performance across various outcomes indicates its practical value in managing COVID-19 patients. Future research should focus on the prospective and multicentric external validation of COVID-COMBI to confirm its predictive accuracy in diverse healthcare settings.

## Figures and Tables

**Table 1:** Assessed risk stratification tools for the prediction of COVID-19 outcomes.

Tool	Original Purpose	Parameters Included
qSOFA	Identify patients with infection at high risk of in-hospital mortality	GCS, respiratory rate, systolic blood pressure
NEWS	Early detection of clinical deterioration (general)	Respiratory rate, SpO_2_, O_2_ supplementation, body temperature, systolic blood pressure, heart rate, AVPU
CURB-65	Predict 30-day mortality in community-acquired pneumonia	Age, confusion, BUN or urea, respiratory rate, systolic blood pressure
4C	Predict in-hospital mortality in hospitalized COVID-19 patients	Age, sex at birth, number of comorbidities ^a^, respiratory rate, SpO_2_ at room air, GCS, urea, CRP
COVID-SEIMC	Predict 30-day mortality in hospitalized COVID-19 patients	Age, sex, age-adjusted SpO_2_, NLR, eGFR, dyspnea
COVID-IRS (NLR)	Predict the need for mechanical ventilation in hospitalized COVID-19 patients	Respiratory rate, SpO_2_/FiO_2_ ratio, LDH, NLR

^a^ The number of comorbidities out of chronic cardiac disease, chronic respiratory disease (excluding asthma), chronic renal disease (estimated glomerular filtration rate ≤ 30), mild to severe liver disease, dementia, chronic neurological conditions, connective tissue disease, diabetes mellitus (diet-, tablet-, or insulin-controlled), HIV or AIDS, and malignancy. Abbreviations: qSOFA: quick Sequential Organ Failure Assessment. GCS: Glasgow Coma Scale. NEWS: National Early Warning Score. SpO_2_: peripheral oxygen saturation. AVPU: Alert, Voice, Pain, Unresponsive scale. BUN: blood urea nitrogen. 4C: 4C mortality score. CRP: C-reactive protein. SEIMC: Sociedad Española de Enfermedades Infecciosas y Microbiología Clínica (Spanish Society of Infectious Diseases and Clinical Microbiology). NLR: neutrophil–lymphocyte ratio. eGFR: estimated glomerular filtration rate. IRS: intubation risk score. FiO_2_: fraction of inspired oxygen. LDH: lactate dehydrogenase.

**Table 2:** New COVID-COMBI score for the risk stratification of severe course in patients hospitalized for COVID-19. For score calculation, all points are added up.

Parameter		COVID-COMBI Points
Age (years)	<50	0
	50–59	2
	60–69	4
	70–79	6
	≥80	7
Sex at birth	Female	0
	Male	1
No. of comorbidities ^a^	0	0
	1	1
	≥2	2
SpO_2_ at room air (%)	≥92	0
	<92	2
GCS	15	0
	<15	2
Urea (mmol/L)	<7	0
	7–14	1
	>14	3
C-reactive protein (mg/L)	<50	0
	50–99	1
	≥100	2
Respiratory rate (breaths/min)	<22	0
	22–29	1
	30–33	2.5
	≥34	3
SpO_2_/FiO_2_ ratio	>200	0
	101–200	2
	≤100	3.5
Lactate dehydrogenase (U/L)	≤200	0
	201–299	1
	300–399	2
	400–499	2.5
	≥500	4
Neutrophil–lymphocyte ratio	<4	0
	4–7.9	1
	8–13.9	2
	≥14	2.5

^a^ The number of comorbidities out of chronic cardiac disease, chronic respiratory disease (excluding asthma), chronic renal disease (estimated glomerular filtration rate ≤30), mild to severe liver disease, dementia, chronic neurological conditions, connective tissue disease, diabetes mellitus (diet-, tablet-, or insulin-controlled), HIV or AIDS, and malignancy. Abbreviations: SpO_2_: peripheral oxygen saturation. GCS: Glasgow Coma Scale. FiO_2_: fraction of inspired oxygen.

**Table 3:** Patient characteristics.

	Overall (*n* = 1051)	Missing (%)
Demographics		
age in years, median (IQR) (range)	65 (54, 79) (19–99)	0
male, *n* (%)	627 (59.7)	0
Comorbidities		
arterial hypertension (%)	481 (45.8)	0
diabetes, *n* (%)	242 (23.0)	0
obesity, *n* (%)	286 (31.0)	12.3
chronic kidney disease, *n* (%)	205 (19.5)	0
chronic liver disease, *n* (%)	59 (5.6)	0
chronic respiratory disease, *n* (%)	201 (19.1)	0
active cancer, *n* (%)	55 (5.2)	0
immunosuppression, *n* (%)	71 (6.8)	0
COVID-19 vaccination status		15.2
not vaccinated, *n* (%)	178 (19.9)	
vaccinated, *n* (%)	101 (11.3)	
no vaccination available, *n* (%)	615 (68.5)	
Admission symptoms		
dyspnea, *n* (%)	483 (46.2)	0.5
cough, *n* (%)	715 (68.5)	0.7
new confusion, *n* (%)	37 (3.5)	0.6
Admission vital signs		
heart rate (bpm), median (IQR)	84 (74, 94)	1.3
systolic blood pressure (mmHg), median (IQR)	134 (120, 149)	0
body temperature (°C), median (IQR)	37.4 (37.0, 38.3)	1.3
fever (body temperature ≥ 38 °C), *n* (%)	397 (38.4)	1.5
respiratory rate (brpm), median (IQR)	21 (18, 25)	0.8
O_2_ saturation at room air (%), median (IQR)	94 (90, 96)	12.0
O_2_ supplementation, *n* (%)	250 (23.8)	0
GCS, median (IQR)	15 (15, 15)	0.3
Admission biomarkers		
leucocytes (×10^9^), median (IQR) ^a^	6.1 (4.6, 8.0)	0.3
neutrophil–lymphocyte ratio, median (IQR) ^b^	5.1 (3.1, 8.5)	5.7
C-reactive protein (mg/L), median (IQR) ^c^	64.5 (28.3, 117.0)	2.0
urea (mmol/L), median (IQR) ^d^	5.6 (4.0, 8.3)	2.4
eGFR (mL/min/1.73m^2^), median (IQR) ^e^	75 (54, 93)	2.1
lactate dehydrogenase (U/L), median (IQR) ^f^	296 (226, 391)	12.8

^a^ Normal range 3.6–10.5. ^b^ Normal range 1–3. ^c^ Normal range < 5. ^d^ Normal range 2.7–6.8. ^e^ Normal range >”90. ^f^ Normal range < 250. Abbreviations IQR: interquartile range. bpm: beats per minute. mmHg: millimeters mercury. C: Celsius. brpm: breaths per minute. O_2_: oxygen. GCS: Glasgow Coma Scale. eGFR: estimated glomerular filtration rate.

**Table 4:** Scores at admission, overall and by patient outcome. Median and interquartile range.

Score	Overall *n* = 1051	Severe Course *n* = 162	In-Hospital Death *n* = 112	Mechanical Ventilation *n* = 74	Missing (%)
qSOFA	1 (0, 1)	1 (1, 1)	1 (1, 1)	1 (1, 1)	0.9
NEWS	4 (2, 6)	6 (4, 9)	6 (3, 8)	7.5 (5, 9)	3.4
CURB-65	1 (0, 2)	2 (1, 3)	2 (2, 3)	1 (0, 2)	3.7
4C	8 (5, 11)	11 (9, 14)	13 (11, 15)	10 (7, 12)	4.9
COVID-SEIMC	8 (5, 15)	13 (8, 19)	17 (11, 21)	8 (5, 11)	7.8
COVID-IRS (NLR)	3 (2, 5)	5 (3, 7)	5 (3, 7)	6 (4, 8)	15.4
COVID-COMBI	11 (8, 14)	16 (12, 20)	17 (14, 20)	14 (11, 19)	18.7

Abbreviations: qSOFA: quick Sequential Organ Failure Assessment. NEWS: National Early Warning Score. 4C: 4C mortality score. SEIMC: Sociedad Española de Enfermedades Infecciosas y Microbiología Clínica (Spanish Society of Infectious Diseases and Clinical Microbiology). IRS: intubation risk score.

## Data Availability

The data presented in this study are available upon reasonable request from the corresponding author. The data are not publicly available due to restrictions in data privacy.

## Appendix A

**Table A1:** Quick Sequential Organ Failure Assessment (qSOFA) [25]. All points are added up.

Parameter		qSOFA Points
Altered mental status (GCS < 15)	No	0
	Yes	1
Respiratory rate (brpm)	<22	0
	≥22	1
Systolic blood pressure (mmHg)	≤100	1
	>100	0

Abbreviations: GCS: Glasgow Coma Scale. brpm: breaths per minute. mmHg: millimeters mercury.

**Table A2:** National Early Warning Score (NEWS) [16]. All points are added up.

Parameter		NEWS Points
Respiratory rate (brpm)	≤8	3
	9–11	1
	12–20	0
	21–24	2
	≥25	3
Peripheral oxygen saturation (%)	≤91	3
	92–93	2
	94–95	1
	≥96	0
Any supplemental oxygen	No	0
	Yes	2
Temperature (°C)	≤35.0	3
	35.1–36.0	1
	36.1–38.0	0
	38.1–39.0	1
	≥39.1	2
Systolic blood pressure (mmHg)	≤90	3
	91–100	2
	101–110	1
	111–219	0
	≥220	3
Heart rate (bpm)	≤40	3
	41–50	1
	51–90	0
	91–110	1
	111–130	2
	≥131	3
AVPU score	A	0
(Alert, Voice, Pain, Unresponsive)	V, P, or U	3

Abbreviations: brpm: breaths per minute. C: Celsius. mmHg: millimeters mercury. bpm: beats per minute.

**Table A3:** Confusion, Urea, Respiratory rate, Blood pressure, Age (CURB-65) score [26]. All points are added up.

Parameter		CURB-65 Points
Confusion	No	0
	Yes	1
BUN (mg/dL)/urea (mmol/L)	≤19/≤7	0
	>19/>7	1
Respiratory rate (brpm)	<30	0
	≥30	1
Blood pressure (mmHg)	systolic ≥ 90 and diastolic > 60	0
	systolic < 90 or diastolic ≤ 60	1
Age (years)	<65	0
	≥65	1

Abbreviations: BUN: blood urea nitrogen. brpm: breaths per minute. mmHg: millimeters mercury.

**Table A4:** 4C mortality score for COVID-19 [24]. All points are added up.

Parameter		4C Points
Age (years)	<50	0
	50–59	2
	60–69	4
	70–79	6
	≥80	7
Sex at birth	Female	0
	Male	1
Number of comorbidities ^a^	0	0
	1	1
	≥2	2
Respiratory rate (brpm)	<20	0
	20–29	1
	≥ 30	2
Peripheral oxygen saturation	≥92	0
on room air (%)	<92	2
GCS	15	0
	<15	2
BUN (mg/dL)/urea (mmol/L)	<19.6/<7	0
	≥19.6–39.2/7–14	1
	>39.2/>14	3
C-reactive protein (mg/L)	<50 mg/L	0
	50–99	1
	≥100	2

^a^ The number of comorbidities out of chronic cardiac disease, chronic respiratory disease (excluding asthma), chronic renal disease (estimated glomerular filtration rate ≤30), mild to severe liver disease, dementia, chronic neurological conditions, connective tissue disease, diabetes mellitus (diet-, tablet-, or insulin-controlled), HIV or AIDS, and malignancy. Abbreviations: brpm: breaths per minute. GCS: Glasgow Coma Scale. BUN: blood urea nitrogen.

**Table A5:** COVID-SEIMC score [23]. All points are added up.

Parameter		COVID-SEIMC Points
Age (years)	<40	0
	40–54	1
	55–64	3
	65–74	5
	75–79	9
	80–84	14
	85–89	15
	≥80	21
Low age-adjusted peripheral	No	0
oxygen saturation ^a^	Yes	2
Neutrophil–lymphocyte ratio	<3.22	0
	3.22–6.33	1
	>6.33	2
eGFR mL/min/1.73 m^2^	≥60	0
	30–59	2
	<30	3
Dyspnea	No	0
	Yes	1
Sex	Female	0
	Male	1

^a^ ≤ 90% for patients aged > 50 years and ≤ 93% for patients aged ≤ 50 years. Abbreviations: eGFR: estimated glomerular filtration rate.

**Table A6:** COVID intubation risk score (COVID-IRS) [22].

Parameter		COVID-IRS Points
Respiratory rate (brpm)	<22	0
	22–29	1
	30–33	2.5
	≥34	3
SpO_2_/FiO_2_ ratio	>200	0
	101–200	2
	≤100	3.5
Lactate dehydrogenase (U/L)	≤200	0
	200–299	1
	300–399	2
	400–499	2.5
	≥500	4
Neutrophil–lymphocyte ratio	<4	0
	4–7.9	1
	8–13.9	2
	≥14	2.5

Abbreviations: brpm: breaths per minute. SpO_2_: peripheral oxygen saturation. FiO_2_: fraction of inspired oxygen.
